# Mobile Applications for Oral Health Promotion in Adolescents: Efficacy, Challenges and Opportunities—A Comprehensive Review

**DOI:** 10.3390/dj14070405

**Published:** 2026-07-03

**Authors:** Joana Fonseca Costa, Carlos Duarte, Luísa Barros, Sónia Mendes

**Affiliations:** 1 Faculdade de Medicina Dentária, Universidade de Lisboa, 1649-003 Lisboa, Portugal; soniaborralho@edu.ulisboa.pt; 2 LASIGE, Faculdade de Ciências, Universidade de Lisboa, 1749-016 Lisboa, Portugal; caduarte@edu.ulisboa.pt; 3 Faculdade de Psicologia, Centro de Investigação em Ciência Psicológica (CICPSI), Universidade de Lisboa, 1649-013 Lisboa, Portugal; lbarros@psicologia.ulisboa.pt

**Keywords:** mHealth, oral health, adolescents, mobile applications, health behaviour, health promotion

## Abstract

**Background/Objectives**: Oral diseases affect around 3.5 billion people worldwide and remain a major public health burden. Adolescence represents a critical stage for establishing lifelong oral health behaviours, particularly given the widespread use of smartphones in this age group. This review aimed to synthesise available evidence on the use of mobile health (mHealth) applications to promote oral health among adolescents, identify app features, evaluate their efficacy in improving oral health outcomes and behaviours, and explore barriers and challenges to their use. **Methods**: A comprehensive review was conducted across PubMed, Scopus, the Cochrane Library, and Web of Science. Studies published between 2010 and 2025 in English, Portuguese, or Spanish that evaluated oral health apps targeting adolescents were included. Data were extracted regarding study design, sample characteristics, app features, clinical and behavioural outcomes, and barriers to app use. Methodological quality was assessed using RoB 2, ROBINS-I, and the JBI checklist, as appropriate. **Results**: Twenty-five studies met the inclusion criteria, comprising 19 randomised controlled trials, 3 qualitative studies, and 3 non-randomised trials. Common app features included educational content, brushing timers, reminders, gamification, and communication with professionals. Most studies demonstrated improvements in plaque and gingival indices, brushing frequency, and oral health knowledge, particularly when apps incorporated behaviour change techniques. However, long-term adherence, usability issues, and lack of regulation remain major limitations. **Conclusions**: mHealth applications show promise as complementary tools for promoting oral health in adolescents. Their success depends on sustained engagement, evidence-based content, user-centred design, and integration with traditional preventive approaches.

## 1. Introduction

According to the latest report from the World Health Organization (WHO), in 2022, oral diseases affected around 3.5 billion people worldwide, making them the most prevalent among more than 300 diseases and conditions affecting humanity [1]. Oral diseases thus carry a heavy global burden [2] and have a very negative impact on an individual’s quality of life [3,4], influencing daily routines and basic needs, and affecting physical, psychological, and social life dimensions [1,5,6].

Dental caries and periodontal disease are the most prevalent oral diseases and are considered preventable, as they are linked to oral hygiene and dietary habits. Therefore, implementing population-based preventive strategies that emphasise the importance of controlling oral biofilm, maintaining a healthy diet, and receiving professional preventive care is highly important [7,8]. The choice of strategy and its implementation must consider the target population, particularly demographic and cultural characteristics, disease distribution, health needs, health literacy, and access to digital technology.

Implementing preventive strategies for adolescents should take into account the specific characteristics of this developmental stage. Adolescence, defined as the age range between 10 and 19 years, is a period characterised by various biological, emotional, and behavioural changes, which can affect both oral and general health [9]. Adolescents constitute a particularly relevant population for oral health research due to the high prevalence of oral diseases observed in this age group, particularly dental caries and gingival inflammation, which remain common despite being largely preventable [10]. During this phase, a range of knowledge and skills essential for developing autonomy and managing interpersonal relationships, as well as elements vital for a successful transition to adulthood, are acquired and refined [11,12,13]. As with other parts of the body, the oral cavity is also impacted by these physical and psychological changes [14,15]. Certain harmful habits, such as smoking, alcohol consumption, and the use of illicit substances, are often first adopted during adolescence and are also reflected in the oral cavity [11]. Conversely, most adolescents exhibit increased concern with physical appearance and aesthetics, aspects that may also influence their perception of and care for the oral cavity. Since adolescence is a period characterised by the gradual acquisition of autonomy and independence [16], it is important to ensure effective reinforcement of oral health education and to provide motivational strategies that promote self-care behaviours and healthy lifestyles. During this stage, implementing positive practices is especially crucial, as behaviours adopted during adolescence tend to persist and significantly influence oral health in adulthood [17].

Another characteristic of this age group is that technology currently exerts a significant influence on their daily routines, with most making extensive use of smartphones [18]. These devices support countless mobile applications (apps) [19], with estimates suggesting that by 2023, 257 billion apps [20] will have been downloaded worldwide, with young people serving as their primary users [21,22,23]. In line with this technological advancement, the healthcare sector has also increasingly adopted technology and apps to maintain and promote healthcare, commonly known as Mobile Health (mHealth) [24]. This concept of mHealth solutions aims at specific objectives, which can be categorised as health promotion and prevention, disease management and monitoring, and remote access to treatment [25]. The number of apps designed to promote oral health and assist with daily care has also kept pace with this rapid technological growth [19,26].

As previously mentioned, young people are the most frequent smartphone users, and adolescence is a vital period for establishing healthy behaviours, which are formed early in life and tend to become ingrained as development continues, making them more resistant to change later in life [27]. Therefore, using apps as educational and preventive tools may be especially relevant for this group. In this context, access to an app that more reliably reinforces the importance of maintaining oral self-care could be an effective and innovative way to supplement consultations and, consequently, improve adolescents’ oral health indicators.

In this context, behaviour change theories provide a useful framework for understanding how such preventive strategies can be translated into effective interventions. These theories help explain why specific features embedded in digital tools, such as self-monitoring, reminders, feedback, and gamification, may support oral health behaviour change by enhancing motivation, engagement, and adherence, ultimately influencing both short- and long-term health outcomes.

A recent review of oral health promotion apps aimed at adults identified a wide range of options but also noted that many are methodologically weak: most lack an explicit theoretical basis, have not been empirically validated, and reveal significant limitations in usability and personalisation [28]. Gaps were also observed in the approach to key risk factors (namely diet, tobacco, and alcohol) and a lack of systematic use of gain-focused messages, which are considered more appropriate for promoting oral hygiene behaviours [28]. Similarly, some authors [19,29] noted that, although apps for promoting oral health offer good functionality, their overall quality is only moderate, mainly due to gaps in the accuracy and comprehensiveness of the information provided to users. However, most existing reviews have focused predominantly on adult populations, and evidence specifically examining the effectiveness of mobile applications in adolescents remains limited.

This review aims to synthesise the available evidence on mobile applications for oral health promotion in adolescents, focusing on their efficacy, challenges, and opportunities. The specific objectives are: (1) to identify and categorise the main features of mobile applications for oral health promotion in adolescents; (2) to assess their efficacy in improving oral health behaviours and outcomes; and (3) to explore challenges and opportunities associated with their adoption and implementation.

## 2. Materials and Methods

A comprehensive narrative review was conducted to synthesise and critically analyse the available literature on the use of apps to promote oral health among adolescents. Although this study incorporates structured elements commonly associated with systematic reviews (e.g., a predefined search strategy, inclusion and exclusion criteria, and a risk-of-bias assessment), it was intentionally designed as a comprehensive narrative review. This approach was chosen to allow for a broader and more flexible exploration of the topic, enabling the inclusion of heterogeneous evidence and the development of a more integrative and interpretative discussion. A narrative synthesis was considered more appropriate to capture the full scope and contextual nuances of the available evidence. Therefore, this review should be interpreted as a narrative review with systematized methods, rather than a formal systematic review.

### 2.1. Study Questions

In light of the objectives outlined above, the research questions for this study were: What are the main features of mobile applications developed to promote oral health in adolescents? What evidence exists regarding the efficacy of these apps in improving oral health indicators? What evidence exists regarding the efficacy of these apps in modifying behaviours related to oral hygiene? What are the main barriers and challenges identified in the adoption and use of mobile apps for promoting oral health among adolescents?

### 2.2. Research Strategy

The literature search was carried out from July 2025 to December 2025, using PubMed, Scopus, the Cochrane Library and Web of Science to ensure the inclusion of all relevant studies on the subject and objectives of this review. An iterative search strategy was employed, with searches being updated and refined throughout the study period to ensure comprehensive coverage of the literature.

The search utilised a carefully selected set of keywords and related terms designed to capture the diversity of existing applications promoting oral health among adolescents. The primary keywords included the following MeSH terms: “Mobile Applications”, “Adolescents”, “mHealth”, “Oral Health”, and “Behaviour Change”. Additional related terms were incorporated, such as “Apps”, “Mobile Apps”, “Teenagers”, “Dental Hygiene”, “Oral Health Promotion”, and “Digital Oral Health”. Boolean operators (AND, OR) were used to combine and refine the search terms.

Two complementary search strategies were conducted to comprehensively identify both oral health promotion applications and those incorporating explicit behavioural change components. The first strategy, used in all platforms (PubMed, Scopus, the Cochrane Library and Web of Science), combined terms related to mobile applications, the adolescent population, and oral health: (“Mobile Applications” OR “Apps” OR “Mobile Apps”) AND (“Adolescents” OR “Teenagers” OR “Youth”) AND (“Oral Health” OR “Dental Hygiene” OR “Oral Health Promotion” OR “Digital Oral Health”). The second strategy built upon the first by adding a behavioural change component to identify digital interventions designed to promote healthy behaviours: (“Mobile Applications” OR “Apps” OR “Mobile Apps”) AND (“Adolescents” OR “Teenagers” OR “Youth”) AND (“Oral Health” OR “Dental Hygiene” OR “Oral Health Promotion” OR “Digital Oral Health”) AND (“Behaviour Change” OR “Behavioral Change”). The search results were refined using database-specific filters. In PubMed, filters were applied to limit studies to the period 2010–2025. In Scopus, filters included English, Portuguese, or Spanish language, Dentistry subject area, article or review document types, and keywords related to adolescents, young adults, oral health, oral hygiene, and health behaviour. In Web of Science, filters restricted the results to articles and reviews published in English, Portuguese, or Spanish. In the Cochrane Library, filters were applied to exclude trial records. In addition, the reference lists of the included articles were screened to identify further relevant publications.

To ensure the scientific relevance of the literature included in this review, specific inclusion and exclusion criteria were applied. The review included empirical primary studies published in English, Portuguese, or Spanish involving adolescents, namely randomised clinical trials, cohort studies, and case-control studies, published between 2010 and 2025. Grey literature was also considered, including technical reports from reputable organisations such as the WHO and the International Dental Federation (FDI). Studies were excluded if they did not primarily focus on oral hygiene behaviour change, did not involve mobile applications for oral health, or were based solely on Short Message Service (SMS) interventions. Systematic reviews and meta-analyses were not included as primary studies in the synthesis; however, they were systematically screened to identify additional eligible primary studies through reference tracking.

### 2.3. Article Selection Process

**Figure 1:**
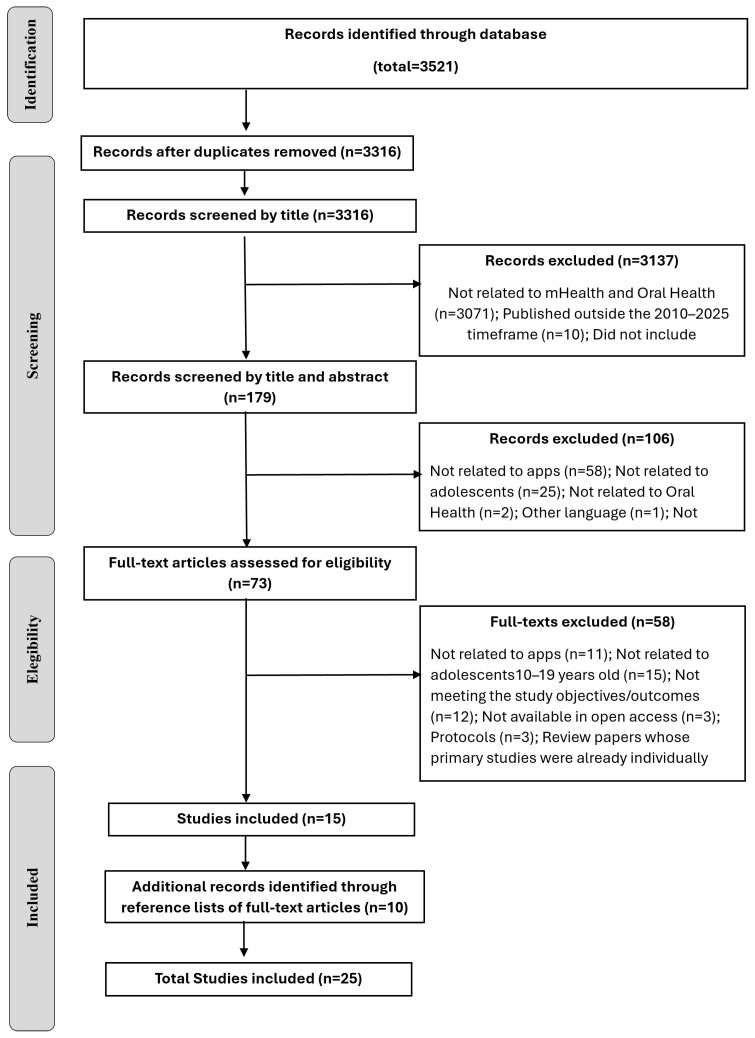
Flow diagram of the study selection process.

After removing duplicate articles, the titles and abstracts of all identified records were reviewed using the previously defined inclusion and exclusion criteria. This stage resulted in the selection of 73 articles for full-text review, of which 25 met the inclusion criteria for this review (Figure 1).

The data extraction process followed a structured approach, focusing on key parameters from each study, including study design, population, app features, outcomes (related to oral health indicators and behaviours), and barriers to app use. The extracted data were organised and synthesised through narrative synthesis, enabling the identification of common trends, emerging themes, and recurring challenges in the adoption of apps to promote oral health among adolescents.

To ensure the reliability and methodological rigour of the included studies and considering the comprehensive nature of this review and the inclusion of multiple study designs, established quality assessment tools commonly used in systematic reviews were applied. Specifically, the Cochrane Risk of Bias Tool (RoB 2) [30] was used for randomised clinical trials; the JBI Critical Appraisal Checklist for Qualitative Research [31] for qualitative studies; and the Risk of Bias in Non-randomised Studies of Interventions (ROBINS-I) [32] for non-randomised intervention studies.

All stages of study screening and data extraction were performed by a single reviewer.

## 3. Results

A total of 25 articles were included, published between 2015 and 2025, of which 19 were randomised controlled trials (RCTs), 3 were qualitative studies, and 3 were non-randomised experimental studies. Table S1 provides a detailed overview of the included studies, summarising the authors, year of publication, study design, objectives, sample characteristics, key features of the evaluated applications, oral health indicators and their respective results, oral hygiene behaviours and associated outcomes, as well as the barriers identified for the adoption and use of these applications.

The included studies, mainly conducted among adolescent populations undergoing orthodontic treatment, highlight common features in mobile applications, namely: toothbrushing timers; educational content delivered through text, images, and videos; reminders (for toothbrushing or dental appointments); personalised feedback (using selfies or Bluetooth-enabled toothbrushes); and gamification elements.

Several randomised controlled trials show significant reductions in oral health indices in app-based intervention groups compared with traditional methods, especially among adolescents with fixed orthodontic appliances. However, some studies find similar clinical improvements between groups with and without app use. On a behavioural level, there is an increase in toothbrushing frequency and duration, flossing habits, and retention of oral health knowledge, particularly when the application is combined with structured oral health education.

The main barriers to app use include declining adherence over time (the novelty effect), technical problems, usability and time constraints, and reluctance to install additional applications.

### Bias Risk Analysis

The methodological quality assessment was carried out according to each study’s design, using specific and widely recognised appraisal tools. The results of this assessment were synthesised narratively and summarised in a dedicated table (Table S2).

Randomised controlled trials were evaluated using the RoB 2 tool, with 12 of 19 studies classified as low risk of bias [33,34,35,36,37,38,39,40,41,42,43,44], and the remaining studies as moderate risk of bias in at least one domain [45,46,47,48,49,50,51]. Table S3 presents the distribution of risk-of-bias assessments across domains, providing an overview of the primary methodological strengths and weaknesses of the included studies.

The three non-randomised intervention studies were evaluated using the ROBINS-I tool, with one study rated as having low risk of bias [52], one as serious risk of bias [53], and one as critical risk of bias [54]. The domain concerning the identification, analysis, and management of confounding factors, as well as their inclusion in statistical modelling, was the most influential in the overall assessment of these studies.

The three qualitative studies were evaluated using the JBI Critical Appraisal Checklist for Qualitative Research [55,56,57]. One study demonstrated moderate methodological quality [55], with some limitations in describing the study context, sampling, and data analysis procedures, while the other two [56,57] exhibited high methodological quality, showing strong coherence between research objectives, study design, data collection, and interpretation of findings.

## 4. Discussion

The integration of digital technologies into healthcare has fostered the development of new strategies to promote health literacy and support behavioural change. In this context, the concept of mHealth is particularly relevant in oral health, as this field continues to face challenges related to adherence to preventive practices and the high prevalence of avoidable oral diseases. The adoption and maintenance of consistent behaviours, such as twice-daily toothbrushing and regular flossing, remain common barriers, especially among adolescents [58,59,60].

Although regular consultations with oral health professionals contribute to disease prevention, the effectiveness of these interventions depends on the inclusion of personalised education strategies and ongoing motivational techniques [61]. Mobile devices, due to their constant proximity to users, enable the implementation of such strategies in everyday contexts where health-related decisions are made and barriers to behavioural change may arise [62]. This emphasises the potential of mHealth interventions to bridge the gap between clinical recommendations and real-life behaviour, especially in younger, digitally engaged populations.

### 4.1. Key Features of Oral Health Promotion Apps

Currently, thousands of applications designed to promote oral health are available in app stores, each offering different functions and aims. Many of these apps are tailored to users’ age groups and specific oral conditions, enabling a more personalised experience that matches their cognitive abilities and interests [63]. For adolescents, the most valued features include disease detection, gamification, educational content, toothbrushing schedules, communication with oral health professionals, reminders and motivational messages, and an interactive, user-friendly design [56,57].

#### 4.1.1. Educational and Informational Features

One of the most common features in oral health apps for adolescents is the provision of educational content [33,35,36,37,38,39,40,42,43,49,55,57]. These apps primarily aim to increase users’ knowledge of appropriate oral hygiene practices and effective strategies for preventing oral diseases, such as dental caries and periodontal conditions.

A 2021 review aimed to identify and evaluate the quality of apps focused on caries prevention [64]. Out of the 562 applications analysed, only 40 met the inclusion criteria. The authors found that most apps focused mainly on oral hygiene behaviours (93%), while aspects such as diet (45%) and fluoride use (42%) were less frequently addressed. Moreover, many applications targeted a single behaviour, such as toothbrushing, rather than adopting a more comprehensive approach that includes multiple determinants of oral health.

Studies relying solely on multimedia educational content (messages, videos, images) showed significant improvements in clinical indicators of gingival inflammation and plaque accumulation (*p* < 0.05) [35,39,43,49], frequency of flossing (*p* = 0.033) [38], and knowledge, attitudes, and self-efficacy related to oral health among adolescents (*p* < 0.05) [39,42,49].

These findings suggest that educational content may contribute to improved oral health outcomes by increasing knowledge, awareness, and self-efficacy. However, as knowledge alone is not always sufficient to sustain behaviour change, educational components may be more effective when integrated with interactive features that reinforce and support daily oral health behaviours.

#### 4.1.2. Toothbrushing Monitoring Features

Toothbrushing monitoring features are also among the most common elements of oral health promotion apps. These usually include timers guiding brushing duration [45,46,54,55] and step-by-step visual or auditory instructions [45,46,48,52,54,65]. Previous studies have demonstrated that integrating such features can enhance adherence to oral hygiene advice, especially among adolescents, by making toothbrushing a more structured and engaging activity [37,45,46,48,54].

For example, the Brush DJ app uses music and a two-minute timer to encourage brushing for the recommended duration, showing a positive impact on adherence to oral hygiene practices, especially among younger populations [55,66]. Similarly, applications such as WhiteTeeth App [37] and My Tooth [48] provide visual brushing instructions. The WhiteTeeth App significantly reduced gingival inflammation (*p* = 0.018) and increased use of fluoride mouthwash (*p* = 0.017) at 6 weeks, as well as plaque accumulation at 12 weeks (*p* = 0.017) [37]. The My Tooth app also showed significant improvements (*p* < 0.05) in knowledge, attitudes, and oral health behaviours compared with baseline and control groups [48].

Beyond improving knowledge, these features may support the formation and maintenance of oral hygiene habits by reducing common barriers to effective toothbrushing and providing real-time behavioural support. Their apparent effectiveness may be explained by the incorporation of behaviour change techniques, such as prompts, cues, and guided instruction, which help translate intentions into consistent actions. This may be particularly important during adolescence, when lifelong health behaviours are still being established.

#### 4.1.3. Habit Monitoring and Reminder Features

There is scientific evidence indicating that reminders and motivational messages can promote adherence to healthy behaviours. A systematic review conducted in 2019 showed that regular reminders increased attendance at routine dental appointments [34].

Some studies report that reminder-based interventions lead to significant reductions in gingival and plaque indices [40,41], although others show no between-group differences or even increases over time [44,50]. A study evaluating the app Healthy Teeth–Tooth Brushing Reminder with timer found significant improvements in the gingival index (*p* = 0.0002) and Turesky-modified Quigley Hein index (*p* = 0.0053) in the intervention group compared with controls [47].

The use of apps integrated with Bluetooth-enabled electric toothbrushes, providing real-time feedback and longitudinal behaviour tracking, has also been associated with sustained improvements in clinical outcomes among adolescents [45,65].

The variability in outcomes suggests that reminders may be most effective when combined with other behaviour change strategies rather than used in isolation. Features such as personalised feedback, progress tracking, and real-time monitoring may strengthen the effect of reminders by increasing user engagement and reinforcing adherence to oral hygiene behaviours. This highlights the importance of considering not only the presence of reminders but also their integration into the overall intervention design.

#### 4.1.4. Communication with Oral Health Professionals

With advances in telemedicine, several apps now offer features that allow users to receive professional advice remotely. These apps feature synchronous communication (such as real-time video or chat) and asynchronous tools (such as messages, photo sharing, and reports), which can help overcome barriers related to time, distance, and access to care [67,68]. Such functionalities support triage and personalised recommendations, enabling professionals to assess the need for in-person intervention and offer tailored feedback [67,68]. The ability to send intraoral photographs or videos allows for immediate professional guidance, thereby enhancing the effectiveness of self-care practices. These digital interactions have been linked to increased motivation and positive behavioural changes, especially among adolescents, who tend to prefer accessible and technology-based methods [33,36,42]. Studies using platforms such as WhatsApp and Telegram reported significant reductions in gingival and plaque indices, along with improvements in brushing frequency and oral health attitudes [33,36]. This highlights the importance of integrating professional support within digital interventions to enhance both credibility and user engagement.

#### 4.1.5. Gamification

Several studies included in this review also incorporated gamification elements [33,36,37,46,52,57]. For example, users of the My Smile app reported higher engagement and enjoyment due to its game-based structure, which strengthened motivation and helped sustain behavioural changes [57].

A scoping review conducted in 2024 concluded that most oral health apps incorporating gamification include evidence-based content and behaviour-change techniques, such as performance feedback and rewards [68]. Gamification structures were found to increase user engagement, improve oral health literacy, and promote attention to preventive behaviours. Participants reported greater satisfaction when learning through interactive, game-based approaches compared with traditional methods [69].

The positive effects of gamification may stem from its ability to increase motivation and engagement through rewards, challenges, and progress tracking. Such features may be especially appealing to adolescents, who are generally more receptive to interactive and rewarding digital experiences. Nevertheless, the extent to which these effects persist remains uncertain, underscoring the need for studies with longer follow-up periods.

### 4.2. Effectiveness of Mobile Applications on Oral Health Outcomes

The widespread use of smartphones makes app-based interventions especially beneficial because of their interactivity, personalisation, accessibility, and cost-effectiveness [70]. These tools can support traditional care by reinforcing clinical instructions, enabling communication with professionals, and offering ongoing access to reliable information [35,46]. Across the included studies, features such as brushing monitoring, real-time feedback, and continuous access to information were consistently linked with better adherence to oral hygiene routines and increased motivation among adolescents [33,36,37,41,42,43,47,49,57,65]. Gamification elements have also demonstrated positive effects on engagement, partly because of the motivational role of social competition [37,71]. However, evidence supporting the effectiveness of gamification in mHealth remains limited and needs theoretically grounded design approaches [72].

Interventions based on behavioural theories, such as self-efficacy, have shown promising results. A randomised controlled trial using the Teeth Care app demonstrated significant improvements in knowledge, attitudes, self-efficacy, and behaviours in the intervention group compared with the control group [42]. Similarly, a pilot study in orthodontic patients found greater reductions in plaque and gingival inflammation when an app was used alongside standard care [50]. Nevertheless, not all studies demonstrate superior clinical outcomes compared with traditional methods. In several cases, improvements occurred in both intervention and control groups, with no statistically significant differences [35,37,38,39,46,49,50,54]. This may be because traditional oral health education is already effective, creating a ceiling effect that limits measurable gains from app-based interventions. Mobile applications may therefore act mainly as complementary tools that reinforce and support behaviour rather than replace conventional approaches. Furthermore, short follow-up periods and variability in study design, intervention characteristics, and outcome measures may also help explain the lack of clear superiority of apps over standard methods. Additionally, the relatively small sample sizes in many studies may have limited the statistical power to detect significant differences between intervention groups.

Additionally, apps may offer advantages for specific populations. Studies involving adolescents with sensory impairments or autism showed improvements in knowledge and clinical outcomes when apps were adapted to their needs [51,52]. This highlights the potential of personalised digital interventions to improve accessibility and inclusivity in oral health promotion.

These findings should be interpreted cautiously, given the methodological limitations and heterogeneity of the included studies. Although most randomised controlled trials showed low overall risk of bias, some studies raised concerns in specific domains, and the non-randomised studies presented more important limitations. In addition, differences in study design, populations, interventions, outcomes, and follow-up periods may help explain the variability of findings across studies. Moreover, a large proportion of the included studies involved adolescents undergoing orthodontic treatment, who are typically seen regularly for clinical follow-up, which may have facilitated adherence and limited the generalisability of the findings to the broader adolescent population.

### 4.3. Challenges and Limitations

Despite their potential, several barriers hinder the sustained use of oral health apps among adolescents. Personal factors, such as a lack of interest in oral health, affect app adoption, with users tending to engage only with applications seen as appealing or relevant [56]. Technical constraints, such as limited storage capacity, connectivity issues, and concerns about data sharing, also pose significant barriers, especially among adolescents using lower-cost devices [36,37,46,56]. Usability issues, repetitive notifications, time constraints, and the novelty effect further decrease long-term engagement [46,57]. Although adolescents recognise apps as credible sources of information, this is often not enough to overcome practical barriers [56]. In addition, real-world use is frequently limited by long-term abandonment after initial adoption, a well-recognised challenge in digital health interventions, often related to declining engagement once novelty decreases.

Moreover, some studies suggest that apps may not be more effective than traditional educational methods, with certain interventions reporting better outcomes using printed materials or face-to-face approaches [53,73].

Despite these limitations, mobile technologies continue to play an increasingly important role in promoting healthy behaviours among digitally connected populations. Early intervention is particularly relevant, as habits developed during adolescence tend to persist into adulthood [74]. Therefore, prolonged exposure to digital reinforcement may be more effective when implemented early, though its success depends on sustained engagement and proper design.

Finally, the lack of regulation in mHealth remains a concern, as many applications are developed without adequate clinical validation [75]. International recommendations, such as those from the FDI, emphasise the importance of safeguarding data, ensuring scientific accuracy, and upholding ethical standards in app development [76].

Overall, the findings of this review show that mobile applications have considerable potential to promote oral health among adolescents, especially when they incorporate a range of behaviour change strategies and are customised to users’ needs. However, their success depends on maintaining engagement, proper design, and integration with traditional preventative methods.

The findings of this review should be interpreted with caution, as the evidence base was characterised by methodological limitations, substantial heterogeneity, and reliance on a single reviewer for study selection and screening. As a narrative review with systematized methods, this approach enabled a broader and more flexible synthesis of heterogeneous evidence; however, it may have introduced selection and interpretation bias and may limit reproducibility compared with fully systematic reviews. Furthermore, differences across studies in design, intervention characteristics, outcome assessment, and follow-up periods complicated direct comparisons and reduced confidence in the consistency of the reported effects. Therefore, the observed benefits of mobile applications should be considered promising, but not definitive.

Future research should focus on longitudinal studies with longer follow-up to assess the sustainability of app-based effects. Larger and more diverse samples are needed to improve generalisability and examine differences across sociodemographic factors, digital literacy, and oral health status. Interventions should be grounded in behavioural theory, with clinically validated content and clearer reporting of components, adherence, and implementation outcomes. Finally, future studies should also evaluate privacy, acceptability, and cost-effectiveness to support safe and scalable implementation in oral health promotion.

## 5. Conclusions

Mobile health applications represent a promising opportunity to promote oral health among adolescents, offering interactive, personalised, and accessible features that can be a useful complement to traditional education and prevention efforts. The increasing variety of available applications includes educational and informational content, structured toothbrushing guidance, habit monitoring, reminders, communication with oral health professionals, integration of artificial intelligence, and gamification. Current evidence suggests that these features may help improve oral health behaviours and reduce plaque accumulation and gingival inflammation, especially when incorporated into interventions based on strong behaviour-change models.

The app’s use represents a promising complementary approach, and its integration with traditional preventive strategies can be well-designed, especially for young people. However, evidence of their superiority over conventional interventions remains inconsistent, and challenges related to long-term adherence, usability, lack of regulation, ethical concerns about data privacy, and limited scientific validation persist. Therefore, these tools should be developed according to high-quality standards, incorporating evidence-based content, user-centred and engaging design, and features that promote sustained use, while actively involving oral health professionals in both their development and clinical implementation.

Future research, based on rigorous methodological designs and including long-term clinical outcome assessment, is essential to consolidate the role of mobile applications as an effective complementary tool in promoting oral health among adolescents.

## Data Availability

No new data were created or analysed in this study. Data sharing is not applicable to this article.

## Supplementary Materials

The following supporting information can be downloaded at: https://www.mdpi.com/article/10.3390/dj14070405/s1, Table S1: Detailed characterisation of the included studies; Table S2: Risk of Bias analysis; Table S3: RCT Risk of Bias (RoB I).

## Abbreviations

The following abbreviations are used in this manuscript:

WHO	World Health Organization
APP	Mobile Application
FDI	International Dental Federation
SMS	Short Message Service
RoB 2	Cochrane Risk of Bias Tool
JBI	Joanna Briggs Institute
ROBINS-I	Risk of Bias in Non-randomised Studies of Interventions
RCT	Randomised Controlled Trial
