# Supplementary material for: Mobile Applications for Oral Health Promotion in Adolescents: Efficacy, Challenges and Opportunities—A Comprehensive Review
Source: Dent J (Basel). 2026 Jul 3;14(7):405. doi: 10.3390/dj14070405 (PMC13408725; doi:10.3390/dj14070405)
Supplement: Supplementary file 1 [file dentistry-14-00405-s001.zip › Table S1.pdf]

Table S1 – Detailed characterisation of the included studies

Study (year)	Design	Study objective	Sample	Main features	Outcomes: oral health indicators	Outcomes: Knowledge, attitudes and behaviours	Barriers / challenges
Zotti et al. (2016) [33]	RCT	Impact of a mobile app on oral hygiene maintenance in adolescents with fixed orthodontic appliances	n=80 fixed appliances ≈13–14 years 2 groups	Educational videos; Selfies share; gamification; Feedback via WhatsApp	Gingival and Plaque Indices lower in app group at 6, 9, 12 months ($p<0.05$ to $p<0.0001$); fewer white spot lesions at 9 and 12 months (($p<0.05$; $p<0.0001$)	Not reported	Not reported
Alkadhi et al. (2017) [34]	RCT	Compare an app-based reminder with verbal instructions	n=44 mean 17 years 2 groups	Reminders to brush three times per day	Plaque and Gingival Indices reduced in the app group from T1 to T2 ($p<0.05$); greater periodontal improvements compared to controls ($p<0.05$).	Not assessed	Not reported
Marchetti et al. (2018) [35]	Cluster RCT	Influence of an app combined with conventional educational methods	n=291 mean 16.1 years 4 groups	Educational messages twice daily (text or video)	Improvement in periodontal disease knowledge in app groups ($p<0.001$); All groups improved Gingival and Oral Hygiene Indices ($p<0.001$)	Better knowledge retention supporting maintenance of good oral hygiene habits	Not reported
Erbe et al. (2019) [45]	RCT	Effectiveness of an interactive power brush with app vs manual brush in orthodontic adolescents	n=59 fixed applicances 13-17 years; 2 groups	Feedback on brushing time; tracking of focus-care areas; performance monitoring, personalisation, and motivational elements	Greater whole-mouth plaque reduction at 2 and 6 weeks (Turesky-modified Quigley-Hein Index) in the interactive group ($p<0.001$)	Brushing time and self-reported motivation increased in the interactive group at follow-up	Not reported
Deleuse et al. (2020) [46]	RCT	Improvement of oral hygiene compliance in orthodontic patients with an app connected to an electric toothbrush	n=38 fixed appliances 12–18 years 2 groups	Monitoring of brushing position, time and pressure; feedback; performance-score gamification; appointment reminders	Plaque and Gingival Indices reduction in both groups ($p \leq 0.0028$), with similar evolution between-group differences; white spot lesion score stable without significant differences	Brushing ≈2 times/day for ≈2 min; app use declined over 18 weeks; moderate perceived increase in brushing motivation.	Drop-off in app use (novelty loss), need for smartphone and stable Bluetooth connection, moderate motivation to continue the use
Scheerman et al. (2020) [36]	RCT	Effectiveness of the WhiteTeeth app in improving oral hygiene in orthodontic patients	n=132 fixed appliances ≈13–14 years 2 groups	Theory-based (HAPA) app; feedback; gamification; reminders; educational content	Gingival bleeding reduced in the intervention group ($p<0.05$) at 6 weeks; Greater dental plaque reduction vs control at 12 weeks:	At 6 weeks: increased fluoride mouthrinse use; no significant effects on behaviour score, brushing frequency/duration, or interproximal brush use	App errors; need a compatible smartphone, willingness to install the app and share data
Scheerman et al. (2020) [37]	RCT	Efficacy of a theory-based Telegram program on oral hygiene behaviour	n=792 mean 15.3 years; 3 groups	Theory-based program (BCT)via Telegram; video education; gamification; feedback; selfies with disclosing solution	Both intervention groups showed significant reductions in Visual Plaque Index and Community Periodontal Index vs control ($p<0.001$)	Increase in brushing frequency in intervention groups, strongest when mothers involved ($p<0.01$)	Need for continuous smartphone/Telegra m access, limiting use in low-access settings
Marchetti et al. (2020) [38]	RCT	Effect of information technologies on increasing dental floss use	n=291 mean 16.1 years 4 groups	Educational messages twice daily (text or video)	Improvements in clinical indices in all groups ($p<0.005$)	Flossing increased in all groups ($p<0.001$), higher floss use in app groups ($p<0.033$)	Not reported

Scribante et al (2021) [39]	RCT	Instagram-based education compared with traditional education on oral health in orthodontic adolescents	n=40 fixed appliances 13-19 years	Instagram images and videos on oral health	Bleeding Index, Modified Gingival Index and Plaque Index improved over 1 month in both groups ($p<0.05$), no differences between-groups	App group showed significant gains in oral health knowledge ($p<0.05$)	Not reported
Bilen et al, (2021) [47]	RCT	Effects of manual and interactive powered toothbrushes (with app) on periodontal status during fixed orthodontic treatment	n=36 12-18 years 2 groups	App-guided brushing with facial recognition, feedback, pressure control, and personalisation	Plaque and Gingival Indices reductions in both groups, greater in the interactive group (PI: $p=0.014$; GI: $p=0.001$); Bleeding on Probing decreased in both ($p<0.001$), with an additional reduction in the interactive group, but no differences between groups.	Not assessed	Not reported.
Rahaei et al. (2022) [48]	Cluster RCT	Effect of a dental-oral health app with traditional education amongst elementary school students	n=158 10-12 years) 2 groups	Brushing guided by images and audio; videos on brushing/flossing; written advice; mascot-based stories	Not reported	App group showed significant improvements in knowledge, attitudes and behaviours vs baseline and vs control ($p<0.05$)	Not reported
Baherimoghadam et al. (2022) [40]	RCT	Effect of reminders on oral hygiene during removable orthodontic treatment	n=58 mean 10 years 2 groups	Text reminders for self-care; educational videos	Lower Gingival and Plaque Indices in the app group at 3 and 6 months ($p<0.05$); slight increase between 3 and 6 months ($p<0.05$); no significant differences in caries index.	Not assessed	Likely novelty effect and declining impact of continuous reminders
Zareban et al. (2022) [49]	RCT	Effect of a Telegram-delivered education program on self-efficacy, beliefs and oral health outcomes	n=160 12-18 years) 2 groups	Educational content with videos to motivate oral self-care	Differences between groups in gingival index after intervention ($p=0.01$)	Differences in self-efficacy, perceived benefits and motivational beliefs ($p=0.01$); no differences in dental cleaning behaviour	Not reported
Lopes dos Santos et al. (2023) [41]	RCT	Effects of an app on adolescents' oral hygiene during fixed orthodontic treatment.	n=8 14-19 years 2 groups	Customizable brushing reminders	Non-significant improvements in Visible Plaque Index and Gingival Bleeding Index (app group)	Not assessed	Not reported
Marashi et al. (2024) [42]	RCT	Self-efficacy-based educational app for promoting oral health behaviours	n=80 girls 13-15 years 2 groups	Oral-health education via text, images and videos	Not reported.	Improvements in knowledge, attitudes, self-efficacy and related behaviours in the app group ($p<0.05$)	Not reported
Bahaa & Selim (2024) [50]	RCT	Effect of a smartphone app as an educational tool and reminder of oral hygiene instructions	n=60 12-19 years 2 groups	Brushing reminders; GIF-based instructions; toothbrush-change reminders	Differences between-group in mean Gingival Index and Modified Quigley-Hein Index (GI: $p=0.0002$; QHTMI: $p=0.0053$) - greatest improvement in smartphone group	Not assessed	Not reported
Fageeh et al. (2024) [51]	RCT	Evaluate a smartphone app for oral health education in blind and deaf adolescents.	n=100 12–18 years 4 groups	Multimedia Arabic sign-language videos; audio-based instructions for blind; video demonstrations for deaf; autoplay guidance	Not assessed	Improvement in knowledge of toothbrushing, interdental aids and nutrition in app group ($p<0.001$)	Not reported

Cimen & Baser (2025) [43]	RCT	Compare traditional vs social-media app motivation strategies for oral hygiene in fixed orthodontic treatment	n=44 mean 15.68 years 2 groups	Educational images and videos; short motivational notifications	Gingival, Bleeding and Plaque Indices improved significantly more in app group (p=0.001)	Not assessed	Not reported
Sembawa et al (2025) [44]	RCT	Effectiveness of an app-based intervention to improve oral hygiene compliance during fixed orthodontic treatment.	n=60 mean 18 years 2 groups	Brushing reminders; brushing calendar for self-recording	Both groups showed significant decreases in Plaque and Gingival Indices (p<0.05); no significant between-group differences	Not assessed	Not reported
Zahid et al (2020) [53]	Quasi-experimental (Non-RCT)	Effect of a mobile app vs lecture-based education on oral health knowledge and behaviours	n=271 mean 16,6 years 2 groups	Music, timer, oral health info, reminders	Not assessed	Improved knowledge, attitudes and behaviour in both groups; lecture group improved twice-daily and 2-min brushing	App perceived as harder to use than lectures
Krishnan et al. (2021) [52]	Interventional (Non-RCT)	Visual pedagogy vs Brush Up app on oral hygiene in adolescents with Autism Spectrum Disorder	n=60 13–17 years 2 groups	3D animated step-by-step brushing, music, mascot, gamification	Plaque and gingival scores improved over time in both groups; no significant differences between visual pedagogy and app	Improved brushing habits and parental perception of adolescents oral health	Not reported
Calderon et al. (2023) [54]	Non-Randomised Pilot Clinical Trial	Effectiveness of a text message to an app on increasing the frequency and duration of tooth brushing	n=90 12-14 years 2 groups	Monitoring of brushing position, time and pressure; feedback; SMS support	Significant reduction of total bacteria and streptococci in both groups (p<0.001); mutans streptococci unchanged	Brushing frequency improved more in SMS-only group than in app+SMS group (p=0.02)	Not reported
Underwood et al. (2015) [55]	Qualitative (questionnaire)	Assess user perception of an oral health app	n=189 7–12 years	Music, brushing timer, oral health education, reminders (dental visit, fluoride use, toothbrush change)	Self-reported improvement in tooth cleanliness (70%) and gingival bleeding (39.3%)	Increased motivation to brush more often and for longer (80%), perceived educational benefits and better adherence.	Not reported
Ab Mumin et al. (2022) [56]	Qualitative focus-group study	Explore adolescents' opinions on using a smartphone application for oral health education and to identify preferred app features	n=77 14 and 16 years	No app tested; preferred features for a future oral health app	Not assessed	Recognising potential benefits for oral-health education and behaviour change, adolescents felt could make it easier to learn key skills and access trustworthy oral-health information compared with general web searches	Limited phone storage and reluctance to install additional apps.
Saari et al. (2025) [57]	Qualitative focus-group study	Acceptability of an app designed to improve oral health knowledge and behaviours	n=23 15-16 years	Educational content; games and rankings; brushing and diet reminders; brushing calendar	Not reported	Adoption of healthy habits, improved brushing frequency and technique, increased oral health knowledge	App not user-friendly, repetitive reminders, lack of time to use the app
