# Supplementary material for: Mobile Applications for Oral Health Promotion in Adolescents: Efficacy, Challenges and Opportunities—A Comprehensive Review
Source: Dent J (Basel). 2026 Jul 3;14(7):405. doi: 10.3390/dj14070405 (PMC13408725; doi:10.3390/dj14070405)
Supplement: Supplementary file 1 [file dentistry-14-00405-s001.zip › Table S2.pdf]

Table S2. Risk of Bias Analysis

Author (Year)	Study Design	Tool	Overall assessment
Zotti et al. (2016) [33]	RCT	RoB 2	Low Risk of Bias
Alkadhi et al. (2017) [34]	RCT	RoB 2	Low Risk of Bias
Marchetti et al. (2018) [35]	RCT	RoB 2	Low Risk of Bias
Erbe et al. (2019) [45]	RCT	RoB 2	Moderate Risk of Bias
Deleuse et al. (2020) [46]	RCT	RoB 2	Moderate Risk of Bias
Scheerman et al. (2020) [36]	RCT	RoB 2	Low Risk of Bias
Scheerman et al. (2020) [37]	RCT	RoB 2	Low Risk of Bias
Marchetti et al. (2020) [38]	Cluster RCT	RoB 2	Low Risk of Bias
Scribante et al (2021) [39]	RCT	RoB 2	Low Risk of Bias
Bilen et al, (2021) [47]	RCT	RoB 2	Moderate Risk of Bias
Rahaei et al. (2022) [48]	Cluster RCT	RoB 2	Moderate Risk of Bias
Baherimoghadam et al. (2022) [40]	RCT	RoB 2	Low Risk of Bias
Zareban et al. (2022) [49]	RCT	RoB 2	Moderate Risk of Bias
Lopes Dos Santos et al. (2023) [41]	RCT	RoB 2	Low Risk of Bias
Marashi et al. (2024) [42]	RCT	RoB 2	Low Risk of Bias
Bahaa & Selim (2024) [50]	RCT	RoB 2	Moderate Risk of Bias
Fageeh et al. (2024) [51]	RCT	RoB 2	Moderate Risk of Bias
Cimen & Baser (2025) [43]	RCT	RoB 2	Low Risk of Bias
Sembawa et al (2025) [44]	RCT	RoB 2	Low Risk of Bias
Zahid et al (2020) [53]	Quasi-experimental (Non-RCT)	ROBINS-I	Serious Risk of Bias
Krishnan et al. (2021) [52]	Interventional (Non-RCT)	ROBINS-I	Low Risk of Bias
Calderon et al. (2023) [54]	Non-Randomized Pilot Clinical Trial	ROBINS-I	Critical Risk of Bias
Underwood et al. (2015) [55]	Qualitative study (questionnaire)	JBI Qualitative	Moderate methodological quality
Ab Mumin et al. (2022) [56]	Qualitative study (focus group)	JBI Qualitative	High methodological quality
Saari et al. (2025) [57]	Qualitative study (focus group)	JBI Qualitative	High methodological quality
