# Supplementary material for: Mobile Applications for Oral Health Promotion in Adolescents: Efficacy, Challenges and Opportunities—A Comprehensive Review
Source: Dent J (Basel). 2026 Jul 3;14(7):405. doi: 10.3390/dj14070405 (PMC13408725; doi:10.3390/dj14070405)
Supplement: Supplementary file 1 [file dentistry-14-00405-s001.zip › Table S3.pdf]

Table S3 – RCT Risk of Bias (RoB I)

Autor (year)	D1	D2	D3	D4	D5	Overall
Zotti et al. (2016) [33]	+	+	+	+	+	+
Alkadhi et al. (2017) [34]	+	+	+	+	+	+
Marchetti et al. (2018) [35]	+	+	+	+	+	+
Erbe et al. (2019) [45]	!	+	+	+	+	!
Deleuse et al. (2020) [46]	+	!	+	+	+	!
Scheerman et al. (2020) [36]	+	+	+	+	+	+
Scheerman et al. (2020) [37]	+	+	+	+	+	+
Marchetti et al. (2020) [38]	+	+	+	+	+	+
Scribante et al (2021) [39]	+	+	+	+	+	+
Bilen et al, (2021) [47]	!	+	+	+	+	!
Rahaei et al. (2022) [48]	!	+	+	+	+	!
Baherimoghadam et al. (2022) [40]	+	+	+	+	+	+
Zareban et al. (2022) [49]	+	!	+	!	+	!
Lopes Dos Santos et al. (2023) [41]	+	+	+	+	+	+
Marashi et al. (2024) [42]	+	+	+	+	+	+
Bahaa & Selim (2024) [50]	+	!	+	+	+	!
Fageeh et al. (2024) [51]	+	!	+	!	+	!
Cimen & Baser (2025) [43]	+	+	+	+	+	+
Sembawa et al (2025) [44]	+	+	+	+	+	+

D1 – Randomization process
D2 – Deviations from the intended interventions
D3 – Missing outcome data
D4 – Measurement of the outcome
D5 – Selection of the reported results

+ Low risk
! Some concerns
- High risk
